# Genetic variation of aggrecanase-2 (*ADAMTS5*) in susceptibility to osteoarthritis

**DOI:** 10.1590/1414-431X20188109

**Published:** 2019-01-10

**Authors:** Xindie Zhou, Lifeng Jiang, Yi Zhang, Junjie Zhang, Dong Zhou, Lidong Wu, Yong Huang, Nanwei Xu

**Affiliations:** 1 Department of Orthopedics, The Affiliated Changzhou No. 2 People's Hospital of Nanjing Medical University, Changzhou, China; 2 Department of Orthopedic Trauma, The Affiliated Changzhou No. 2 People's Hospital of Nanjing Medical University, Changzhou, China; 3 Department of Orthopedic Surgery, The Second Affiliated Hospital, Zhejiang University School of Medicine, Hangzhou, China

**Keywords:** ADAMTS5, Polymorphism, Osteoarthritis, Co-expression analysis

## Abstract

Aggrecanase-2 (*ADAMTS5*) gene is responsible for aggrecan degradation that may contribute to cartilage destruction in a mouse osteoarthritis (OA) model. We aimed to investigate the effects of *ADAMTS5* gene polymorphisms on OA risk in a Chinese population. A total of 300 OA patients and 300 controls were recruited and their genotypes for *ADAMTS5* gene rs226794 and rs2830585 polymorphisms were determined using a custom-by-design 48-Plex single nucleotide polymorphism Scan™ kit. *ADAMTS5-*associated genes were identified by co-expression analysis and their functions were investigated by Gene Ontology and Kyoto Encyclopedia of Genes and Genomes analyses. Bioinformatics analysis showed that *ADAMTS5* was significantly related to the components, structural constituent, and organization of the extracellular matrix. The rs2830585 polymorphism, but not rs226794 polymorphism, was significantly associated with an increased risk of knee OA. Stratified analysis further confirmed this significant association in patients at age ≥55 years. In conclusion, the *ADAMTS5* rs2830585 polymorphism may be involved in the development of knee OA by destroying the extracellular matrix, but this finding should be further confirmed by larger studies.

## Introduction

Osteoarthritis (OA) is the most common form of arthritis and a major socioeconomic burden (1). The incidence of OA increases with aging and is higher among women, especially after 50 years of age (2). OA of the hips and knees tends to cause severe disability requiring surgical intervention (3). Notable risk factors of OA include age, obesity, gender, smoking, genetics, diet, and occupation (4). Identification of OA-associated genes can help identify the biological mechanisms of OA (5).

OA is characterized by degeneration of articular cartilage and changes in periarticular and subchondral bones (6). The degeneration is attributed primarily to uncontrolled destruction of the extracellular matrix (ECM), including the proteoglycan aggrecan and type II collagens (7). Aggrecanase-mediated aggrecan degradation plays an important role in OA development (8,9). Two cartilage aggrecanases [aggrecanase-1 (ADAMTS4) and aggrecanase-2 (ADAMTS5)] have been identified, which are both very efficient in cleaving soluble aggrecan at the Glu (373)-Ala (374) site (10). A surgical mouse OA model shows that *ADAMTS5* ablation can essentially eliminate cartilage erosion and fibrous overgrowth (11). Therefore, *ADAMTS5* may play a crucial role in OA development.

The function of *ADAMTS5* may be influenced by two non-synonymous single nucleotide polymorphisms (SNPs) by altering the amino acid sequence of the protein. The association of *ADAMTS5* gene variants [rs226794 (P692L in exon 7) and rs2380585 (R614H in exon 5)] with OA development has been studied in various populations (12–14), but not in East China. Therefore, the present study was conducted to evaluate the effects of *ADAMTS5* gene polymorphisms on OA risk in an East Chinese Han Population.

## Material and Methods

### Study subjects

A total of 300 knee OA patients (test group) and 300 controls (control group) were recruited from the Affiliated Changzhou No. 2 People's Hospital of Nanjing Medical University (China) and the Second Affiliated Hospital of Medical College, Zhejiang University (China) between October 2013 and November 2017. Knee OA was diagnosed in accordance with the criteria of the American College of Rheumatology (1987) (15): primary OA with any symptom and radiographic sign of OA according to the Kellgren-Lawrence (K-L) grading system. Patients with post-traumatic or post-septic arthritis, inflammatory arthritis, or malignant or chronic illness were excluded. The 300 controls were selected from patients attending orthopedic clinics of the same hospitals for treatment of trauma at the time of sampling. Any subject with doubtful diagnosis was excluded. The functional or symptomatic status of patients was assessed using Lequesne functional index. Pain was evaluated by the visual analogue scale (VAS), a pain measure scale. Controls were selected from the patients attending the general surgery and orthopedics clinics of the two hospitals at the time of sample collection. A questionnaire was designed to collect from cases and controls the general information [e.g., age, sex, body mass index (BMI)] and clinical data [e.g., erythrocyte sedimentation rate (ESR) and C-reactive protein (CRP)] of OA.

This study was approved by the Institutional Ethics Committees of the two hospitals. Written informed consent was obtained from each subject.

### Co-expression analysis and SNP selection

*ADAMTS5* coexpressing genes were identified using co-expressed gene database (COXPRESdb) (http://coxpresdb.jp/) and their protein interaction networks were constructed using STRING (https://string-db.org/). The most relevant functions of these genes were identified via Gene Ontology (GO) and Kyoto Encyclopedia of Genes and Genomes (KEGG) analyses.

Linkage data were searched from Ensembl (http://ensembl.org/index.html) and processed on Haploview (https://www.broadinstitute.org/haploview/haploview).

The tag SNPs were selected on Haploview, in which a threshold of r^2^>0.8 was applied in the pairwise correlation, and SNPs with a minor allele frequency <10% were excluded. Functional predictions were performed on SIFT (http://sift.jcvi.org/www/SIFT_dbSNP.html).

### DNA extraction and genotyping

Venous blood (2 mL each) was sampled in tubes containing ethylenediamine-tetraacetic acid (EDTA) and stored at –80°C before use. Genomic DNA was extracted using a QIAamp DNA blood mini kit (Qiagen, Germany), and the concentration and purity were estimated using NanoDrop (Thermo Electron Corp., USA) at two absorbance wavelengths of 260 and 280 nm. Genotyping was done by matrix-assisted laser desorption/ionization time-of-flight mass spectrometry (MALDI-TOFMS) using a MassARRAY system (Sequenom, USA). Completed genotyping reactions were spotted onto a 384-well spectroCHIP (Sequenom) using a MassARRAY nanodispenser (Sequenom) and analyzed by MALDI-TOFMS. Genotype calling was done in real time with MassARRAY RT 3.1 (Sequenom) and analyzed on MassARRAYTyper 4.0 (Sequenom). For quality control, 10% of randomly-selected samples were analyzed repeatedly.

### Enzyme-linked immunosorbent assay (ELISA)

Serum *ADAMTS5* levels of OA patients were measured using the human *ADAMTS5* ELISA kit (Biorbyt, UK), according to the manufacturer's recommendations. Absorbance was read at 450 nm using a microplate reader (TECAN INFINITF*F50, Switzerland). *ADAMTS5* concentration could be determined using the standard curve. The detection range of this kit is 15.6-1000 ng/mL. All samples were determined by the same investigator, who was blind to the clinical situation.

### Statistical analysis

Hardy-Weinberg equilibrium (HWE) was assessed using goodness-of-fit χ^2^ tests to investigate deviation between observed and expected frequencies among controls. Clinical data were compared between groups by the unpaired Student's *t*-test, while qualitative data were compared by the chi-squared test. All genotypes for the two polymorphisms evaluated were divided and analyzed in three distinct genetic models, according to a study by Clarke et al. (16): 1) genotype distribution model (wild-type *vs* heterozygous *vs* mutated); 2) dominant genetic model (wild-type *vs* heterozygous+mutated), and 3) recessive genetic model (mutated *vs* wild-type *vs* heterozygous+wild-type). Odd ratios (ORs) and 95% confidence intervals (CIs) were calculated to evaluate the associations between *ADAMTS5* gene polymorphisms and OA risk by logistic regression analyses. All statistical analyses were performed on SAS 9.1.3 (SAS Institute, USA) with the significant level at P<0.05.

## Results

### Bioinformatics analysis

**Figure 1:**
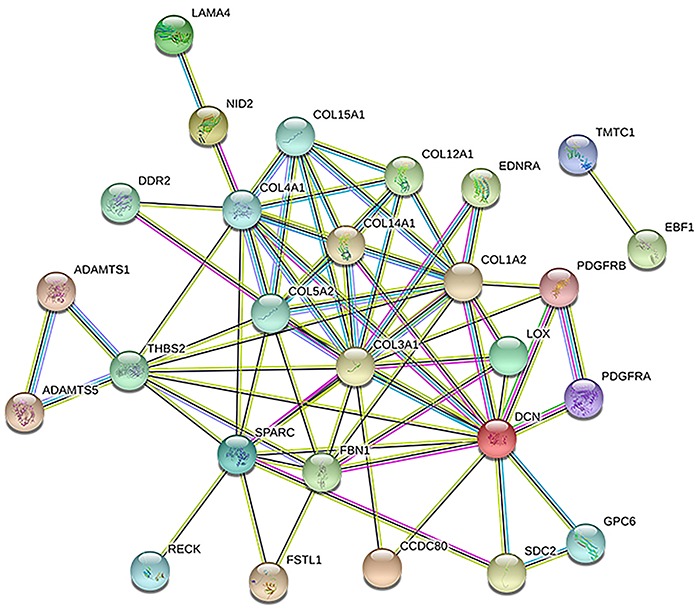
Protein interaction network of *ADAMTS5* co-expression gene.

**Figure 2:**
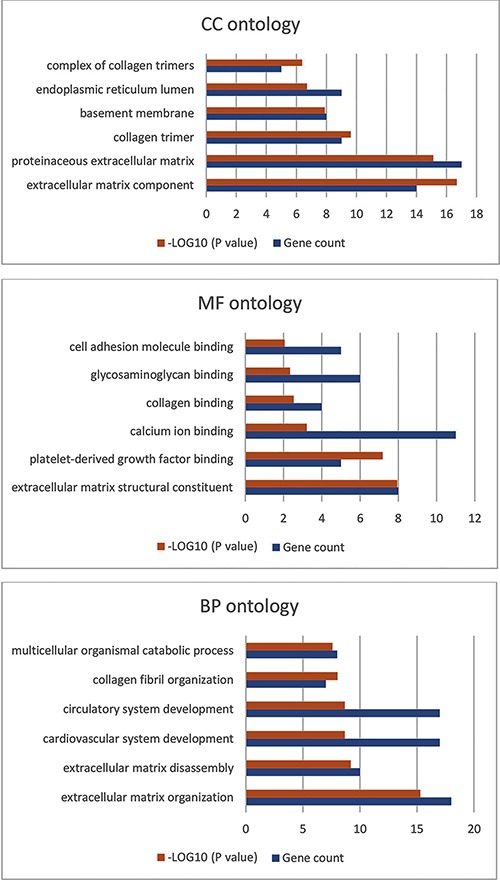
Bar plot of representative Gene Ontology analysis of *ADAMTS5* co-expression gene. CC: extracellular matrix (ECM) component; MF: ECM structural constituent; BP: ECM organization.

The protein-protein association networks co-expressed with *ADAMTS5* are shown in Figure 1. The five genes most related to *ADAMTS5* were ADAM metallopeptidase with thrombospondin type 1 motif 1 (ADAMTS1), fibrillin 1 (FBN1), laminin alpha 4 (LAMA4), protocadherin 18 (PCDH18), and decorin (DCN). LAMA4-integrin signaling contributes to clustering in human osteoarthritic chondrocytes, which is a morphological sign of OA (17). Figure 2 shows the GO enrichment results of these genes. Annotation results indicate these genes are correlated with ECM component (CC), ECM structural constituent (MF), and ECM organization (BP) terms and the protein digestion and absorption pathway. *ADAMTS5* may be involved in OA development through ECM.

**Figure 3:**
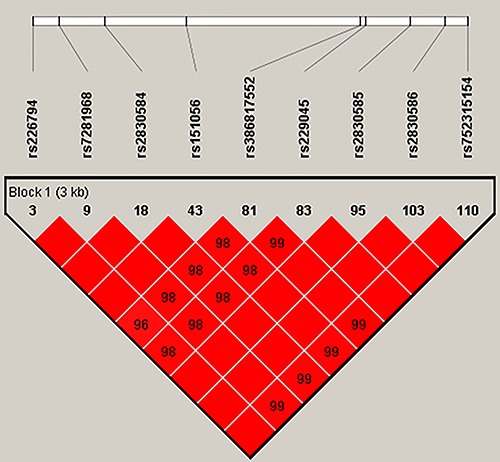
Linkage disequilibrium of the 9 SNPs in *ADAMTS5* gene.

The 9 tagger SNPs screened out on Haploview 4.2 are shown in Figure 3. The relevant parameters were set as follows: HW P-value cutoff=0.05; min genotype=75%; Max# Mendel error=1; minimum allele frequency=0.1. The 9 tagger SNPs included 6 intronic SNPs, 2 missense SNPs, and 1 unknown SNP. SIFT analysis showed the rs226794 polymorphism caused Leu > Pro at amino acid position 692, decreasing the risk of OA. However, rs2830585 was predicted to be deleterious for ADAMTS5 protein function (Table 1).

**Table 1:** Functional single nucleotide polymorphisms (SNP) selection from the 9 tag SNPs.

SNP	Chr pos (hg38)	Ref/Alt	dbSNP func annot	Amino acid change	Amino acid	Prediction (homologs)	Score (homologs)	Median info (homologs)
Rs226794	21:26930036	G/A	missense	L692P	Leu	Damaging	0.02	3.22
					Pro	Tolerated	1	3.22
Rs7281968	21:26930239	T/G	intronic					
Rs2830584	21:26930587	G/A	intronic					
Rs151056	21:26931204	G/A	intronic					
Rs386817552	21:26932519	N/A	N/A					
Rs229045	21:26932555	G/A	intronic					
Rs2830585	21:26932893	C/T	missense	R614H	Arg	Tolerated	1	3.21
					His	Damaging	0	3.21
Rs2830586	21:26931158	T/G	intronic					
Rs752315154	21:26933328	T/A	intronic					

Chr pos: chromosome position.

### Characteristics of subjects

Baseline characteristics are reported in Table 2. The patients were slightly younger and consisted of more females than the controls (P=0.329; 0.719). BMI data were significantly different between groups (P<0.001). The majority of OA cases belonged to K-L grade 2 or 3. We also included several clinical parameters (such as VAS and Lequesne index) to investigate the OA patients' clinical conditions. The observed genotype frequencies of rs226794 in the control group followed the HWE (P=0.997). The genotype distributions of rs2830585 were not significantly different between groups (P=0.961).

**Table 2:** Patient demographics and risk factors for knee osteoarthritis.

Variable	Cases (n=300)	Controls (n=300)	P
Age (years)	58.2±9.1	59.0±9.2	0.329
Sex			
Male	85 (28.3%)	89 (29.7%)	0.719
Female	215 (71.7%)	211 (70.3%)	
Body mass index	26.5±3.3	24.1±3.5	<0.001
CRP, mg/L	24.8±14.1		
ESR, mm/h	21.4±12.6		
VAS	8.1±2.7		
Lequesne index	14.2±2.5		
Kellgren-Lawrence grading			
1	18 (6.1%)		
2	128 (42.6%)		
3	98 (32.7%)		
4	56 (18.6%)		

Data are reported as number and percentage or mean±SD (*t*-test or chi-squared) test) CRP: C-reactive protein; ESR: erythrocyte sedimentation rate; VAS: visual analogue scale.

### Associations between *ADAMTS5* polymorphisms and OA risk

Table 3 shows the genotype and allele distributions for *ADAMTS5* gene variants in both groups. No significant deviation from HWE was found for two SNPs in the controls (P=0.998 for rs226794, P=0.961 for rs2830585, respectively). None of the five models showed any significant association between OA risk and rs226794 polymorphism. The TT genotype of rs2830585 polymorphism was significantly associated with a 1.95-fold increased risk of OA compared with the CC genotype (TT *vs* CC: OR, 1.95; 95%CI, 1.03 3.71; P=0.041). Similarly, the CT+TT genotype was significantly associated with an increased risk of OA (CT+TT *vs* CC: OR, 1.45; 95%CI, 1.05-2.00; P=0.024). After adjusting for gender, age, and BMI, the results were still significant. Furthermore, the rs2830585T allele increased the risk of OA by 39% compared with the C allele (T *vs* C: OR, 1.39; 95%CI, 1.07-1.79; P=0.013).

**Table 3:** Logistic regression analysis of associations between *ADAMTS5* gene polymorphisms and risk of osteoarthritis.

Genotype	Cases^a^ (n=300)		Controls^a^ (n=300)		Hardy-Weinberg equilibrium	OR (95%CI)	P	Adjusted OR (95%CI)^b^	P
	n	%	n	%					
Rs226794 G/A					0.998				
GG	203	67.7	182	60.7		1.00			
GA	86	28.7	101	33.7		0.76 (0.54,1.08)	0.131	0.78 (0.54,1.13)	0.191
AA	9	3.0	14	4.7		0.58 (0.24,1.36)	0.210	0.64 (0.26,1.59)	0.335
GA+AA	95	31.7	115	38.4		0.74 (0.53,1.04)	0.081	0.76 (0.53,1.09)	0.140
GG+GA	289	96.4	283	94.4		1.00			
AA	9	3.0	14	4.7		0.63 (0.27,1.48)	0.288	0.69 (0.28,1.71)	0.426
G allele	492	82.0	465	77.5		1.00			
A allele	104	17.3	129	21.5		0.76 (0.57,1.02)	0.064		
Rs2830585 C/T					0.961				
CC	145	48.3	172	57.3		1.00			
CT	126	42.0	109	36.3		1.37 (0.98,1.92)	0.068	1.41 (0.99,2.02)	0.060
TT	28	9.3	17	5.7		**1.95 (1.03,3.71)**	0.041	**2.07 (1.05,4.10)**	0.037
CT+TT	154	51.3	126	42.0		**1.45 (1.05,2.00)**	0.024	**1.50 (1.06,2.11)**	0.021
CC+CT	273	90.3	281	93.6		1.00			
TT	28	9.3	17	5.7		1.71 (0.91,3.19)	0.093	1.79 (0.92,3.47)	0.087
C allele	416	69.3	453	75.5		1.00			
T allele	182	30.3	143	23.8		**1.39 (1.07,1.79)**	0.013		

Genotyping was successful in 298 cases and 297 controls for rs226794, and 299 cases and 298 controls for rs2830585. ^b^Adjusted for sex, age, and body mass index. Bold values are statistically significant (P<0.05).

**Figure 4:**
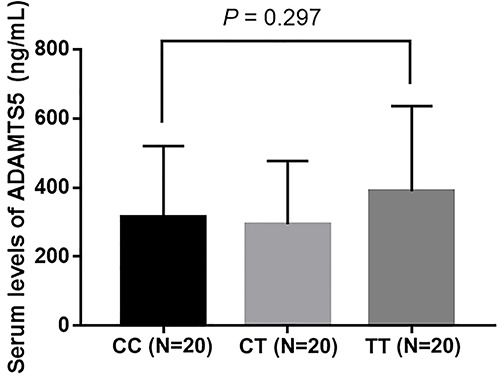
Histogram of serum *ADAMTS5* levels in patients with OA. Data are reported as means±SD (*t*-test).

Haplotypes were established through the use of two SNPs. The distribution of the haplotype frequency of the two polymorphisms for the OA patients and healthy controls are presented in Table 4. There was no significant difference between OA patients and controls with regard to haplotype frequencies of GC, AC, and GT (P>0.05). Stratified analyses according to sex and age are illustrated in Table 5. For subjects at age ≥55 years, *ADAMTS5* rs2830585 polymorphism was significantly associated with an increased risk of OA in the additive model (TT *vs* CC: OR 3.34; 95%CI, 1.38-8.56; P=0.012). Significance was also present in the dominant and recessive models. However, the subgroup analysis of sex showed no significant association. We also investigated the clinical parameters in the different genotypes of rs2830585 polymorphism (Table 6). There was no significant effect on OA risk with regard to BMI, CRP, ESR, VAS, Lequesne index, and K-L grading. Our results indicated that serum *ADAMTS5* levels of the TT genotype were higher than that in the CC genotype, but the result was not significant (Figure 4). This indicated that amino acid changes (Arg>His) may have no effect on *ADAMTS5* protein function and the SIFT prediction may be wrong.

**Table 4:** Estimated haplotype number and relative frequencies for the two *ADAMTS5* variants (rs226794 and rs2830585).

Haplotype	OA	Control	OR (95%CI)	P
GC	263 (0.438)	265 (0.442)	0.99 (0.81,1.22)	0.942
AC	82 (0.137)	109 (0.182)	0.75 (0.55,1.02)	0.070
GT	149 (0.248)	118 (0.197)	1.26 (0.97,1.65)	0.087

OA: osteoarthritis. Chi-squared test.

**Table 5:** Stratified analyses between *ADAMTS5* gene polymorphisms and the risk of osteoarthritis.

Variable	*ADAMTS5* rs2830585 (case/control)			TT *vs* CC OR (95%CI); P value	TT+CT *vs* CC OR (95%CI); P value	TT *vs* CT+CC OR (95%CI); P value
	CC	TC	TT			
Sex						
Male	49/47	30/39	5/3	2.20 (0.78-6.23); 0.138	1.21 (0.66-2.21); 0.542	2.14 (0.80-5.72); 0.131
Female	96/125	96/70	23/14	1.81 (0.76-4.32); 0.182	1.60 (0.95-2.69); 0.078	2.61 (1.08-6.33); 0.415
Age (years)						
<55	46/52	48/35	10/5	1.07 (0.41-2.82); 0.890	1.09 (0.61-1.94); 0.766	1.02 (0.41-2.51); 0.972
≥55	99/120	78/74	18/12	3.34 (1.30-8.56); **0.012**	1.80 (1.05-3.11); **0.034**	2.61 (1.08-6.33); **0.034**

Bold values are statistically significant (P<0.05, chi-squared test).

**Table 6:** Comparison of studied data according to *ADAMTS5* genotypes in all osteoarthritis (OA) cases.

*ADAMTS5* rs2830585	OA (n=300)			P
	CC (n=145)	AG (n=126)	GG (n=28)	
BMI (kg/m^2^, mean±SD)	26.65±3.15	26.40±3.57	26.56±3.06	0.823
ESR (mm/h, mean±SD)	21.26±11.04	21.51±14.23	21.76±13.11	0.725
CRP (mg/L, mean±SD)	25.73±14.45	24.45±14.05	22.63±11.56	0.504
VAS (mean±SD)	8.12±2.60	8.04±2.60	8.50±3.29	0.711
Lequesne index (mean±SD)	13.96±2.49	14.37±2.54	14.43±2.51	0.342
K-L grading (III+IV / I+II, n (%))	75 (51.7%) / 70 (48.3%)	65 (51.6%) / 61 (48.4%)	13 (46.4%) / 15 (53.6%)	0.870

BMI: body mass index; ESR: erythrocyte sedimentation rate; CRP: C-reactive protein; VAS: visual analogue scale; K-L: Kellgren-Lawrence. The *t*-test and chi-squared test were used for statistical analyses. Genotyping was successful in 299 OA patients for rs2830585.

## Discussion

Cartilage consists of a relatively small amount of chondrocytes embedded in abundant ECM, which contains numerous macromolecules, especially collagen fibrils and the large aggregating proteoglycan aggrecan (18). When OA occurs, the degradation of ECM macromolecules surpasses the synthesis, eventually leading to total or partial cartilage erosion (19). *ADAMTS5* could cleave the Glu373-Ala374 bond in the inter-globular domain of aggrecan (20). A murine model of surgically-induced OA shows *ADAMTS5* deficiency could diminish aggrecan loss and cartilage erosion (20). Here, we aimed to investigate the function of *ADAMTS5* through analyzing the enrichment of its co-expressed genes and found that these genes were significantly associated with the components, structural constituent, and organization of the ECM. These genes were also significantly correlated with platelet-derived growth factor binding, calcium ion binding, and collagen binding, which play important roles in OA development.

Two missense SNPs (rs226794 and rs2830585) in *ADAMTS5* were predicted to be deleterious for *ADAMTS5* protein function using the SIFT database. The associations between *ADAMTS5* gene polymorphisms and OA risk have been investigated in three studies, but with conflicting findings (12–14). Rodriguez-Lopez et al. (12) firstly evaluated the association between *ADAMTS5* rs226794 polymorphism and OA risk in 4 Caucasian groups, and found that this polymorphism decreased the risk of knee OA in the Santiago and Thessaly groups (872 cases and 974 controls), but not in the Oxford and Corunna groups. No significant association between *ADAMTS5* rs2830585 polymorphism and OA risk was found in the Santiago group (12). Gu et al. (13) reported that the rs2830585 polymorphism, but not the rs226794 polymorphism, was significantly associated with a decreased risk of OA in 420 OA patients compared with 312 controls. The significant association also held true for cervical OA, but not knee, lumbar, or hand OA (13). Canbek et al. (14) revealed that neither *ADAMTS5* rs226794 nor rs2830585 polymorphism was linked to susceptibility to knee OA in a Turkish population (80 cases and 95 controls). Here, we validated the association between two *ADAMTS5* variants and OA risk in a Chinese population including 300 cases and 300 controls. It was found that rs2830585 polymorphism, but not rs226794 polymorphism, conferred susceptibility to knee OA. This discrepancy may be attributed to sample sizes and ethnicity-dependent effects. Sample sizes in three other studies were relatively small, and thus their findings may be underpowered. The A allele frequency of Asians was higher than that of Caucasians (0.47 *vs* 0.09). We hypothesize that genetic heterogeneity, clinical heterogeneity, different genotyping methods, and random errors may also be potential reasons for different findings between Asians and Caucasians.

There is no significant association between *ADAMTS5* rs226794 polymorphism and OA risk in any single population (Santiago, Thessaly, Oxford, Corunna, China, and Turkey) (12–14). Rodriguez-Lopez et al. (12) found that this polymorphism decreased the risk of knee OA when combined with Santiago and Thessaly. A possible explanation is that the associations among SNPs in genes associated with OA were greatly affected by the number of participants in the study.

Several studies revealed that *ADAMTS5* rs2830585 polymorphism was not linked to susceptibility to OA among Caucasians (12,14). Two Chinese studies evaluated the association between this SNP and OA risk, but with opposite results. The present study shows the A allele of rs2830585 polymorphism increased the risk of OA, while Gu et al. (13) found it decreased the risk of OA. We hypothesize that environmental factors (e.g., geographic location) impact the genotype distribution of rs2830585 polymorphism. Moreover, eating habits vary geographically in China, which may also explain the above findings. Notably, our study is the first to find a significant association between *ADAMTS5* rs2830585 polymorphism and the risk of knee OA. Gu et al. (13) found TT genotype of rs2830585 decreased the risk of cervical OA, which may be partly attributed to the inherent heterogeneity of disease progression in different types of OA. The rs2830585 polymorphism causes Arg>His at amino acid position 614 when the nucleotide changed from C to T. Paired amino acid converting enzyme 4 (PACE4) was identified as a proprotein convertase responsible for activation of aggrecanases (*ADAMTS4* and *ADAMTS5*) (21). We hypothesized that amino acid change alters the spatial structure, making it easier to cleave by PACE4, which in turn increases the expression of *ADAMTS5*. The activation of *ADAMTS5* conferred susceptibility to OA. Considering that the rs2830585 polymorphism may contribute to developmental issues that later manifest as OA, we will conduct long-term follow-up of different genotypes in the OA group and control group to observe the effect of genetic factors on OA pathogenesis.

Several possible limitations need to be addressed. First, the population of this hospital-based study may not be representative of the general population. Second, the sample size, which is not particularly small, is still insufficient to obtain significant results. Third, our results may be affected by confounding factors such as alcohol drinking and smoking. Fourth, the two variants genotyped here do not completely cover the whole *ADAMTS5* gene. Fifth, this was a retrospective study and we could not provide follow-up data.

In conclusion, *ADAMTS5* affected the OA development through ECM, and the *ADAMTS5* gene rs2830585 polymorphism was a genetic contributor to risk of knee OA. These findings may contribute to biomarker development for early detection and risk stratification, but should be confirmed by further studies in larger populations.
